# Endovascular Recanalization of Aortic Isthmus Atresia with an “Electrified Wire Technique”

**DOI:** 10.1177/15266028231206996

**Published:** 2023-10-23

**Authors:** Kugarajah Arulrajah, Konstantinos Spanos, Giuseppe Panuccio, Thomas Gandet, Carsten Rickers, Tilo Kölbel

**Affiliations:** 1 Department of Vascular Medicine, German Aortic Center, University Medical Center Hamburg-Eppendorf, Hamburg, Germany

**Keywords:** thoracic aorta, aortic isthmus atresia, coarctation, congenital heart defects, stent-graft, thoracic endovascular aortic repair, descending thoracic aorta, endovascular treatment

## Abstract

**Purpose:**

For aortic coarctation in adults endovascular repair is the treatment of choice with an acceptable safety profile. Aortic isthmus atresia is a related condition with a complete occlusion of the aorta not allowing catheterization across the isthmus. This technical note describes a recanalization of an aortic isthmus atresia using radiofrequency with an “electrified wire technique.”

**Technique:**

A guidewire was selectively denuded of PTFE (polytetrafluoroethylene) at the distal end and was placed through a catheter distal to the aortic isthmus atresia. The denuded end of the wire was clamped to an electrosurgery pencil. By pushing the wire toward a tulip-snare, which was placed as a target proximal of the occlusion via left trans-brachial access, and shortly activating of the electrosurgery pencil the electrified wire recanalized the occlusion and was snared and used to guide implantation of a balloon-expandable covered stent.

**Conclusion:**

The electrified wire puncture technique can be used to recanalize adult aortic isthmus atresia after failed conventional attempts.

**Clinical Impact:**

The electrified wire technique offers an off-the shelf option to modify standard guidewires for the use with radiofrequency to cross a complete aortic isthmus occlusion after failed conventional attempts. This new technique may be applied also in other situations like dissection flap fenestration, transcaval access and similar.

## Introduction

Coarctation of the aorta accounts for approximately 3% to 5% of congenital heart defects.^1^ Aortic isthmus atresia is a subvariant of coarctation and a rare congenital anomaly with clinical features similar to coarctation of the aorta.^2^ Surgical therapy of coarctation is the treatment of choice for newborns and infants. For children and adolescents, endovascular repair increasingly represents an alternative therapeutic option,^3^ using various types of uncovered and covered stents.^4^

For adults with newly diagnosed aortic coarctation or recurrent coarctation after prior open surgical repair, endovascular repair is safe and effective preferably using balloon-expandable covered stents (BCS).^5–7^ However, endovascular treatment can be prevented in cases of complete occlusion at the level of the aortic isthmus. The presence of this atresia of the aorta can be hard to identify on preoperative cross-sectional imaging and may be verified by periprocedural digital subtraction angiography (DSA), and when the aortic isthmus cannot be catheterized. Previous reports have described a recanalization technique using a transseptal needle.^8,9^

Another technique is a recanalization using radiofrequency with the 0.035 inch PowerWire (Baylis Medical, Canada), which is FDA-approved for totally occluded peripheral vessels of 3 mm and more.^10^ The Nykanen radiofrequency wire (Baylis MedComp Inc, Montreal, Canada) is similar to the PowerWire and was successfully used in Europe.^11^ In this technical note, we describe an alternative technique to recanalize an aortic isthmus atresia using an electrified guidewire based on our experience with the BASILICA technique (Bioprosthetic or native aortic scallop intentional laceration to prevent coronary artery obstruction) described by Khan et al.^12^

## Technique

### Case Presentation

**Figure 1.**
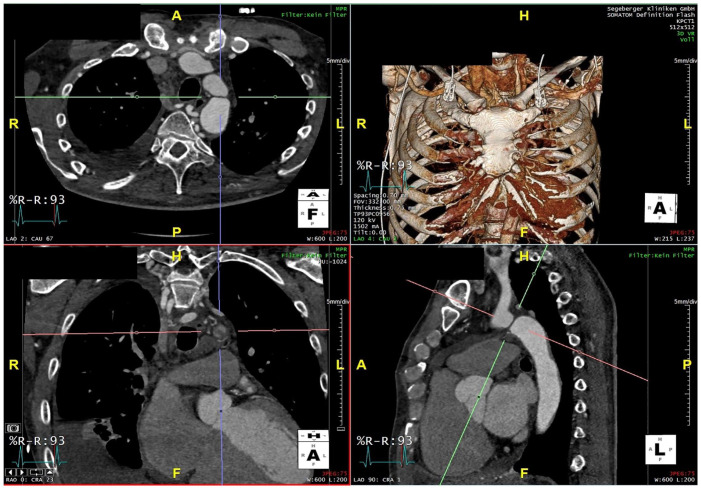
Preoperative computed tomography angiography (CTA) with aortic coarctation.

A 55 year old male patient was treated at an outside hospital for arterial hypertensive crisis and tachyarrhythmia. Arterial hypertension and atrial flutter had not been known yet. Successful ablation of typical atrial flutter was performed. Due to the reduced left ventricular function, coronary angiography was attempted, but it was not possible to access the coronary arteries from a retrograde transfemoral access. A computed tomography angiography (CTA) revealed a typical appearance of an aortic coarctation with a suspicion of complete occlusion of the aorta as no contrast-enhanced connection could be identified between the proximal and distal aortic stump (Figure 1). Typical signs for abdominal angina or claudication were not described by the patient. The systolic pressure gradient from upper to lower extremity was 55 mm Hg. Endovascular repair using a BCS was planned to treat the aortic coarctation, and for the case of unsuccessful conventional catheterization, recanalization with an electrified guidewire was planned.

### Technique

The BASILICA technique (Bioprosthetic or native aortic scallop intentional laceration to prevent coronary artery obstruction) describes the technique of radiofrequency perforation of porcine pericardial leaflet of a bioprosthetic aortic valve to enable transcatheter aortic valve implantation after previous surgical aortic valve replacement protecting proximal coronary artery ostia.^12^ This technique uses continuous duty radiofrequency energy delivery that constantly heats tissue until it vaporizes to perforate or cut through heterologous pericardial tissue. We have recently applied this technology to cheese-wire a dissection membrane in complex thoracoabdominal aortic repair.^13^

**Figure 2.**
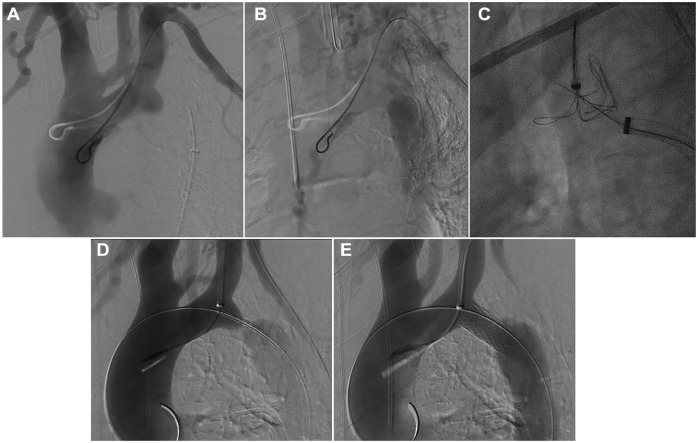
(A) Digital subtraction angiography during the case, which shows the aortic isthmus atresia. (B) Another digital subtraction angiography during the case, which shows the aortic isthmus atresia. (C) Fluroscopy during the case, which shows the electrified wire puncture of the aortic isthmus atresia. (D) Digital subtraction angiography during the case, which shows the angioplasty of the occlusion with an Advanta V12 12 mm × 41 mm. (E) Another digital subtraction angiography during the case, which shows the angioplasty of the occlusion with an Advanta V12 12 mm × 41 mm.

The procedure was performed in a hybrid operation room under general anesthesia and systemic heparinization with a target activated clotting time of 250 to 350 seconds. After percutaneous left brachial access, a pigtail catheter was placed in the aortic arch. Repeated DSA of the aortic arch in multiple projections confirmed a complete occlusion of the aorta at the aortic isthmus (Figure 2A and B). Using percutaneous access to the right common femoral artery, a 14F, 80 cm steerable Fustar sheath (Lifetech Scientific, Shenzhen, China) was placed distal to the aortic isthmus. Attempts of conventional catheterization using different catheters and wires were not successful.

A 7F 55 cm Flexor Ansel sheath was placed from the left brachial access in the proximal left subclavian artery (LSA) and a 7F EN-Snare (MeritMedical, South Jordan, UT, USA) was placed in the proximal aortic stump to serve as a target for the retrograde puncture with an electrified wire and to allow snaring of the wire. A 300 cm, 0.014 inch Astato XS20 guidewire (Asahi-Intecc, Tokyo, Japan) was selectively denuded of PTFE (polytetrafluoroethylene) on a short segment at the distal end with a conventional scalpel and clamped to an electrosurgery pencil (70 W). This wire was positioned through a 5F 100 cm Lindh catheter (Cordis, Dublin, Ireland) within the 14F Fustar sheath. The Lindh catheter was equipped with a Tuohy-Borst adapter to allow for continuous flushing with 5% dextrose as a nonionic liquid promote charge concentration in the target tissue and prevent blood coagulation during energy delivery.^14^ Using the assembly with steerable sheath and Lindh catheter, the wire-end was pointed toward the opened EN-Snare using multiple projections. Orthogonal projections were especially used to ensure the right position of the wire to reduce the risk of aortic perforation as described in other studies.^15^ By pressing the yellow button on the electrosurgical pencil, the cutting mode was activated and the wire easily moved through the tissue into the proximal aortic stump within the wings of the EN-Snare (Figure 2C). A femoro-brachial wire was established and the Lindh-catheter easily passed into the proximal stump allowing an exchange for an extra-stiff double-curved Lunderquist wire (Cook Medical, Bjæverskov, Denmark). The 14F Fustar sheath was advanced with its dilator into the aortic arch. An Advanta V12 12 × 41 mm (Getinge AB, Göteborg, Sweden) BCS was positioned at the atresia and deployed after sheath-removal (Figure 2D and E). Post dilatation with a 16 mm high-pressure Atlas Gold balloon Catheter (BD, Franklin Lakes, NJ, USA) and a compliant 32 mm Coda balloon (Cook Medical, Bjæverskov, Denmark) was done to achieve better wall apposition.

**Figure 3.**
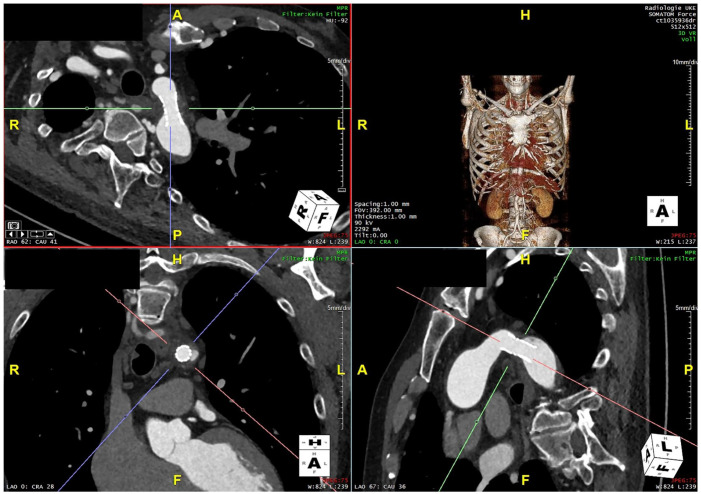
Postoperative Computed tomography angiography with a wide aortic isthmus.

Final angiography showed a good result without any signs of wall injury or dissection. The post-op CTA confirmed the good outcome (Figure 3). The patient was discharged on the fourth postoperative day in good condition with unchanged anti-hypertensive medication and normal blood pressure of 120/80 mm Hg.

## Discussion

The use of radiofrequency current has been described for recanalization of several peripheral and central vascular occlusion including subclavian vein occlusion, nonmalignant superior vena cava occlusion, anterior and posterior tibial arteries, common iliac vein, and superior vena cava, pulmonary artery, and re-entry for ostial right coronary artery chronic total occlusion.^14^ We recently described the “powered cheese-wire technique” in a case of branched endovascular aortic repair (BEVAR) of a complex thoracoabdominal dissecting aneurysm in which access to a renal artery arising from the false lumen was gained by longitudinally cutting the dissection flap using electrical current on a noninsulated through-and-through wire segment with a “flying V” configuration.^13^

In a previous study, an aortic isthmus atresia was recanalized in a 16 year old patient using radiofrequency with a PowerWire (Baylis Medical) and a BCS.^10^ In another study, 4 adult patients aged between 32 and 63 years with an aortic isthmus atresia were successfully treated with a Nykanen radiofrequency wire (Baylis MedComp Inc, Montreal, Canada) and a covered Cheatham-Platinum stent.^11^

With the described “electrified wire technique,” a standard guidewire can be utilized for radiofrequency use in fenestration and recanalization inspired by the BASILICA technique. This new technique offers an off-the-shelf option to modify standard guidewires for the use with radiofrequency and may be applied for other situations like dissection flap fenestration, transcaval access, and similar.

## Conclusion

The electrified wire puncture technique can be used to cross a complete aortic isthmus occlusion after failed conventional attempts.
